# Unveiling the piezoelectric nature of polar α-phase P(VDF-TrFE) at quasi-two-dimensional limit

**DOI:** 10.1038/s41598-017-18845-2

**Published:** 2018-01-11

**Authors:** Jun Qian, Sai Jiang, Qijing Wang, Shushu Zheng, Shuya Guo, Chang Yi, Jianpu Wang, Xinran Wang, Kazuhito Tsukagoshi, Yi Shi, Yun Li

**Affiliations:** 1 National Laboratory of Solid-State Microstructures, School of Electronic Science and Engineering, Collaborative Innovation Center of Advanced Microstructures, Nanjing University, Nanjing, 210093 P. R. China; 2 International Center for Materials Nanoarchitectonics (WPI-MANA), National Institute for Materials Science (NIMS), Tsukuba, Ibaraki 305-0044 Japan; 3 Key Laboratory of Flexible Electronics and Institute of Advanced Materials, Jiangsu National Synergistic Innovation Center for Advanced Materials, Nanjing Tech University, Nanjing, 211816 P. R. China

## Abstract

Piezoelectric response of P(VDF-TrFE), which is modulated by the dipole density due to the polarization switching on applying an electric field, allows it act as the fundamental components for electromechanical systems. As proposed since the 1970s, its polar α-phase is supposed to yield an enhanced piezoelectric activity. However, its experimental verification has never been reported, hampered by a substantial challenge for the achievement of a smooth, neat α-phase film. Here, we prepare ultrathin crystalline α-phase P(VDF-TrFE) films on the AlO_x_/Al-coated SiO_2_/Si substrates via a solution-based approach at room temperature. Thus, we unveil the piezoelectric nature of the polar α-phase P(VDF-TrFE) at a quasi-two-dimensional limit. The obtained values of the relative morphological deformation, the local effective piezoelectric coefficient, and the electric field-induced strain reach up to 37 pm, −46.4 pm V^−1^, and 4.1%, respectively. Such a robust piezoelectric response is even higher than that of the β-phase. Besides, the evolution of piezoelectricity, which is related to the piezoelectric properties of two polarization states, is also studied. Our work can enable the exploration of the prospective applications of polar α-phase P(VDF-TrFE) films.

## Introduction

Piezoelectricity, which roots from an inter-transformation between the electrical polarization and the mechanical deformation, is a fascinating property for broad microelectronic applications^1–3^. In particular, poly(vinylidene fluoride-co-trifluoroethylene) (P(VDF-TrFE)), a representative piezoelectric polymer, has been regarded as an ideal candidate for electromechanical systems by virtue of its structural tunability, good solubility, and thus simplicity in processing^4–8^. To achieve a respectable piezoelectric property is essential for further application of P(VDF-TrFE) in advanced mechatronics engineering. So far, piezoelectric P(VDF-TrFE) films are commonly prepared by mechanically and electrically orientating the molecules, and the resulting crystals generally adopt a β-phase conformation^9–12^. Nevertheless, the polar α-phase proposed since the 1970s is overlooked, and its piezoelectric properties have never been experimentally verified^13–15^. Notably, an improved piezoelectric activity is speculated to be yielded for a polar α-phase P(VDF-TrFE), which arises from the optimal dipole ordering with its alternating polymer backbone arrangements^16,17^. Besides, to study the electrical polarization-modulated electromechanical coupling behaviours of the polar α-phase P(VDF-TrFE) is also greatly important to elucidate the effects of specific molecular conformations on the intrinsic piezoelectric properties; also, confirming the piezoelectricity of the polar α-phase P(VDF-TrFE) will be the most compelling evidence for the dipole model^17^. However, microstructural reorganization often leads to an α- to β-phase conversion^8,18^, owing to the thermodynamic instability of the α-phase film^4^. Such a long-standing challenge for the fabrication of a smooth and neat α-phase P(VDF-TrFE) film has severely hindered the achievement of its piezoelectric behaviours. Consequently, it is of great significance to explore the piezoelectric properties of the solution-processed polar α-phase P(VDF-TrFE) films.

Herein, we report the fabrication of ultrathin α-phase P(VDF-TrFE) films with ultrathin thicknesses on AlO_x_/Al-coated SiO_2_/Si substrates at room temperature. Therefore, we unveil the piezoelectricity of the polar α-phase P(VDF-TrFE) at a quasi-two-dimensional limit. The relative morphological deformation, the local effective piezoelectric coefficient *d*_zz_, and the maximum electric field-induced strain of the polar α-phase P(VDF-TrFE) yield values of 37 pm, −46.4 pm V^−1^, and 4.1%, respectively. The piezoelectric activity is even higher than that of the β-phase. Besides, we study the evolution of polarization-patterned piezoelectric domains, which is related to the piezoelectric properties of two polarization states. Our results should facilitate further study of the piezoelectric properties relevant to the molecular structures, thus providing a promising avenue that explores potential applications of the polar α-phase P(VDF-TrFE) for piezoelectronics.

## Results

### Fabrication and crystalline properties of ultrathin α-phase P(VDF-TrFE) films

**Figure 1:**
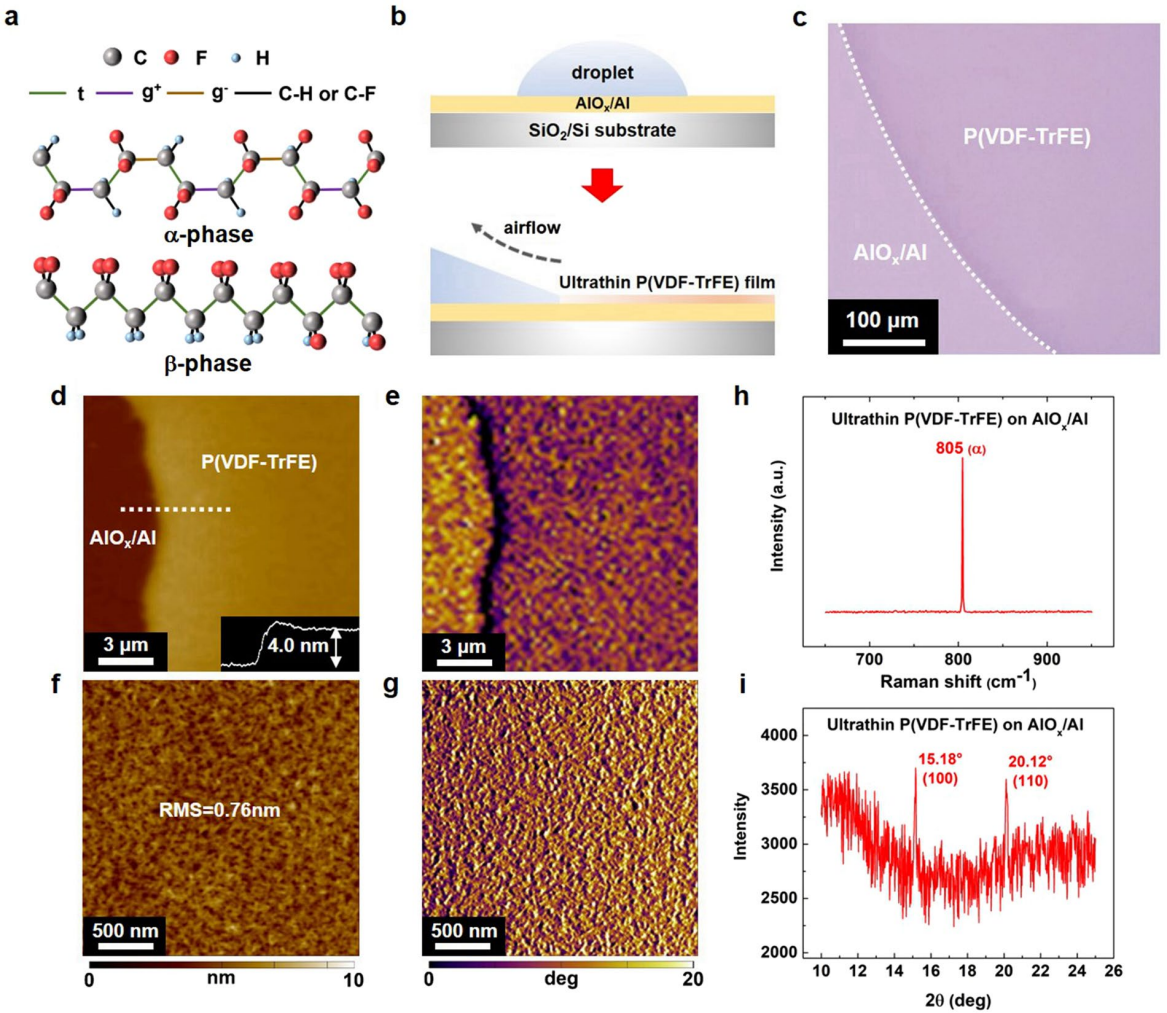
Fabrication and crystalline properties of the α-phase P(VDF-TrFE) films. (**a**) Molecular structure of the α- and β-phase P(VDF-TrFE). (**b**) Schematic of the solution-coating process. A droplet of P(VDF-TrFE) intermixed solution was drop-cast onto the AlO_x_/Al surface, and dragged by the airflow. And a scalable P(VDF-TrFE) film growth was deposited with no post-annealing treatment. (**c**) Optical micrograph of the P(VDF-TrFE) film. (**d**,**e**) AFM height and phase images of a P(VDF-TrFE) film. (**f**,**g**) AFM surface topography and phase images of a P(VDF-TrFE) film. (**h**) Raman spectra of the sample in Fig. 1c, clearly showing the signal of the α-phase. (**i**) 2*θ* scan image taken from the GI-XRD measurement, indicating that the P(VDF-TrFE) films are composed of the α-phase conformation.

The two most common polymorphs of P(VDF-TrFE) are shown in Fig. 1a. The crystalline α-phase is formed by -(CH_2_-CF_2_)_n_-(CHF-CF_2_)_m_- chains in a distorted trans-gauche^+^-trans-gauche^−^ (tg^+^tg^−^) conformation, and the β-phase is composed of an all-trans planar zigzag structure (tttt)^19^. In our previous report, we have used the antisolvent crystallization technique to deposit two-dimensional small-molecule crystalline films^20,21^. To prepare the P(VDF-TrFE) films on the atomically smooth AlO_x_/Al substrates with the root-mean-square (RMS) roughness of 0.24 nm (Supplementary Fig. S1), we used *N,N*-dimethylformamide (DMF) as the major solvent and a small amount of *p*-anisaldehyde as the antisolvent. A schematic of the solution-coating process is illustrated in Fig. 1b. The solution was drop-cast onto an AlO_x_/Al-coated SiO_2_/Si substrate. And then, a mechanical pump generated airflow that dragged the droplet to move rapidly on the substrate. In the optical micrograph illustrated in Fig. 1c, the deposited P(VDF-TrFE) film is extremely uniform over a large area of ~400 μm.

The P(VDF-TrFE) film morphology was characterized by using atomic force microscopy (AFM). As shown in Fig. 1d, an obtained film is as thin as only ~4.0 nm, a thickness comparable to that of a two-molecular-monolayer (2-ML) Langmuir-Blodgett (LB) film^22^. The region of the AlO_x_ and the ultrathin P(VDF-TrFE) is easily distinguished because of the apparent colour contrast in the phase image (Fig. 1e). Besides, the obtained P(VDF-TrFE) exhibits a polycrystalline phase with a grain size of ~30 nm. And it has an extremely smooth surface with the root-mean-square (RMS) roughness of only 0.76 nm (Fig. 1f). In addition, Fig. 1g shows that the ultrathin film is homogenous over a large area, exhibiting as a quasi-two-dimensional feature. Thus, the as-prepared ultrathin P(VDF-TrFE) films deposited with the antisolvent crystallization technique show continuous film crystallinity without requiring any post-annealing treatment.

We further performed the micro-Raman spectroscopy and the grazing-incident X-ray diffraction (GI-XRD) characterizations to demonstrate the achievement of α-phase for our P(VDF-TrFE) film. As shown in Fig. 1h, a sharp Raman peak at 805 cm^−1^ is observed in the spectrum of the ultrathin P(VDF-TrFE) film on an AlO_x_/Al surface, which is assigned to the crystalline α-phase with an alternating trans-gauche conformations^23^. In addition, we recorded the Raman spectra before and after a thermal-annealing treatment at 60 °C for 10 min. The shifted peak position reveals that the crystalline α-phase is intrinsically metastable (Supplementary Fig. S2). Similar α- to β-phase transitions have been previously observed through an electrospinning process^8^. Furthermore, as shown in Fig. 1i, the GI-XRD results show the peaks with remarkable signals for the (100) and the (110) diffractions at 2*θ* = 15.18° and 2*θ* = 20.12°, respectively, indicating that our P(VDF-TrFE) is crystallized with the α-polymorph structure^24^. Besides, the stable α-phase P(VDF-TrFE) at ambient conditions should be attributed to the specific structures (e.g., head-to-head defects, chain ends), as proposed previously^25^. As further increasing the film thickness to ~15 nm, we observe that the film is a mixture of the α- and β-phases owing to a destabilizing effect of the TrFE monomers^26^ (Supplementary Fig. S3).

### Piezoelectric features of polar α-phase P(VDF-TrFE) films

**Figure 2:**
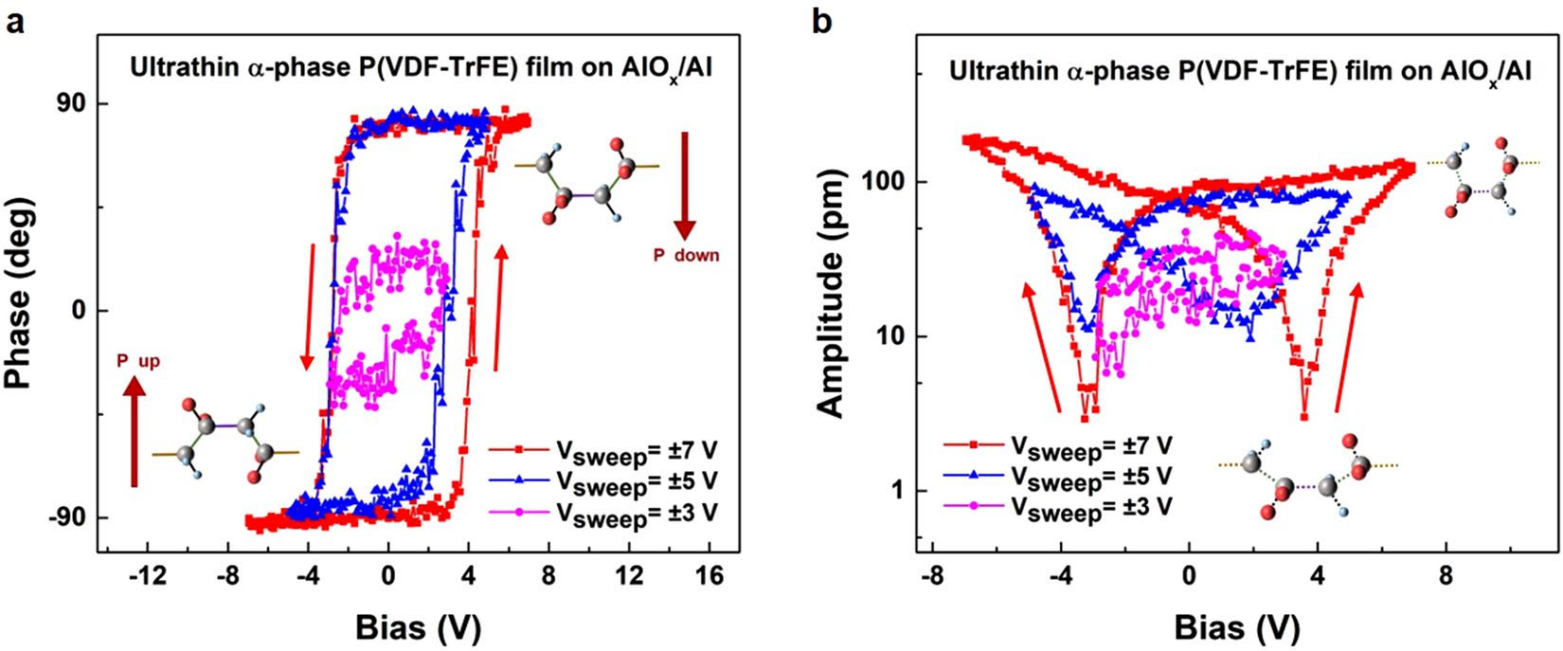
Piezoelectric hysteresis loops of the quasi-two-dimensional α-phase P(VDF-TrFE) films on AlO_x_/Al substrates. (**a**) Local PFM phase curves with an unambiguous 180° phase shift. The insets show the molecular structures corresponding to the two polarization states. (**b**) Local PFM amplitude curves with an expected classical butterfly shape. The insets show the stretched and compressed configurations of the α-phase P(VDF-TrFE). Hysteresis loops are recorded with three different sweep voltages of ±3 V, ±5 V, and ±7 V; the sweep direction is indicated by the arrows.

Encouraged by the high-quality α-phase P(VDF-TrFE) films, we used the high-sensitivity piezoresponse force microscopy (PFM) to characterize their piezoelectric nature. A typical piezoelectric response relies on the switchable crystalline dipoles. When applying an electric field on the P(VDF-TrFE) films, the change of dipole density owing to the polarization reversal induces the mechanical deformation^27^. Thus, such switchable dipoles were characterized using a PFM tip-generated electric field onto our pristine P(VDF-TrFE) films. As shown in Fig. 2, we obtain a full set of hysteresis loops for the local PFM phase and amplitude signals versus the voltage. The phase and amplitude represent the polarization orientation and piezoelectric strain, respectively. At a low sweep bias (*V*_sweep_ = ±3 V), both the phase and amplitude loops begin to show hysteresis. The sweep bias is then increased to ±5 V, and we obtain unsaturated local hysteresis loops. The full phase and amplitude loops with the formed polar phase are achieved after increasing the maximum applied sweep bias to ±7 V. The local coercive voltages, *V*_c_, the threshold switching voltages of the electrical polarization orientations, amount to approximately +3.7 and −3.2 V. This coercive voltage is much lower than that of bulk P(VDF-TrFE) crystals^4^, thus indicating that the reduction in structural dimension is favourable for achieving a low-voltage polarization reversal. Moreover, we used micro-Raman spectroscopy to confirm that the obtained piezoelectric features originated from the polar α-phase P(VDF-TrFE) rather than a β-phase one (Supplementary Fig. S4). Similar to the electroforming of the polar α-PVDF^16^, our PFM electrical poling only led to the formation of the polar α-phase P(VDF-TrFE).

**Figure 3:**
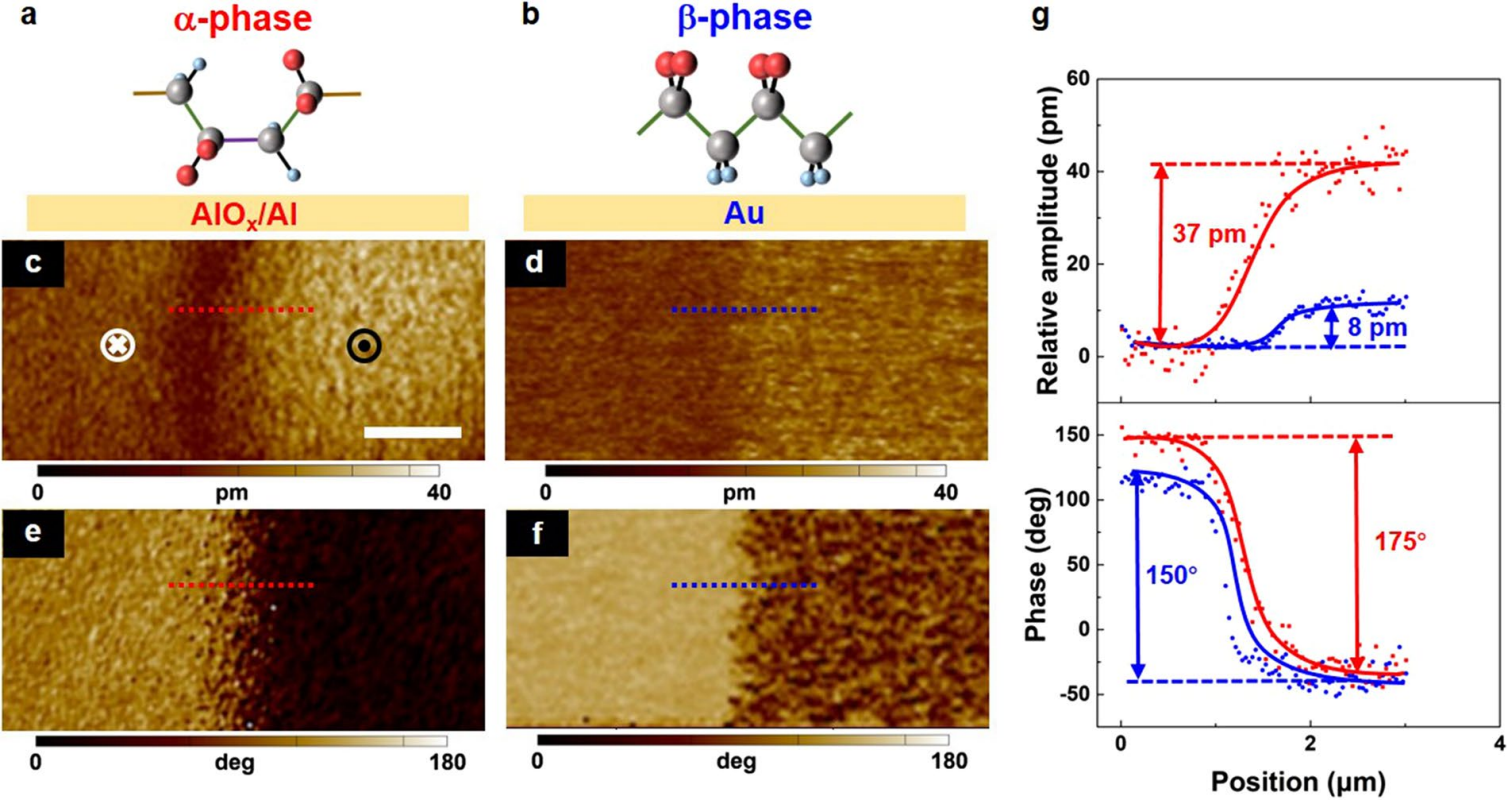
Comparison of the piezoelectric PFM images between ultrathin polar α-phase and β-phase P(VDF-TrFE) films. (**a**,**b**) Schematics of the α- (**a**) and β-phase (**b**) P(VDF-TrFE) on AlO_x_/Al and Au substrates, respectively. (**c**,**d**) The PFM out-of-plane amplitude images of the ultrathin polar α- (**c**) and β-phase (**d**) films. (**e**,**f**) The PFM phase images of the ultrathin polar α- and β-phase films. All PFM images were recorded after a domain was written with a biased conductive tip. Scale bar: 2 μm. (**g**) The profiles of the relative amplitudes and the phase contrasts that are correspond to the dotted lines in the PFM images.

Next, the piezoelectric properties of the polar α-phase P(VDF-TrFE) were compared with that of the β-phase by using a resonance-enhanced PFM. Ultrathin piezoelectric β-phase P(VDF-TrFE) films were fabricated on the Au-coated SiO_2_/Si substrates (Supplementary Figs S5–6). The schematic of the polar α- and β-phase P(VDF-TrFE) on AlO_x_/Al and Au substrates are shown in Fig. 3a and b, respectively. The ultrathin P(VDF-TrFE) films with different polymorphs were both written with a biased PFM tip. The left- and right-hand regions of the scanning domain were poled using different voltages (+7 V and −7 V) for the definition of the downward and upward polarizations, respectively (Fig. 3c–f). This process was followed by a PFM tip scanned over the entire pre-poled areas. Figure 3g shows the profiles of the relative amplitudes and the phase contrasts that are correspond to the dotted lines in the PFM images. Because the tip resolution is 50 nm and the grain size is 30 nm, the PFM tip spatially takes two or three grains in the scan direction. Thus, the neighbouring polarized areas exhibit a transition region rather than a sudden change. As shown in the upper half of Fig. 3g, the polar α-phase P(VDF-TrFE) exhibits a notable mechanical strain of ~37 pm (the relative amplitude is imaged in Fig. 3c). And it is much higher than that of the β-phase (~8 pm, the PFM imaging of the relative amplitude is shown in Fig. 3d). Also, as described below, our polar α-phase films yield higher local effective piezoelectric coefficient *d*_zz_ (−46.4 pm V^−1^) and maximum electric field-induced strain (4.1%) than the β-phase ones. It thus reinforces the stronger negative piezoelectric response in the quasi-two-dimensional polar α-phase P(VDF-TrFE) films^26^.

Additionally, the polar α-phase P(VDF-TrFE) exhibits a large phase contrast of ~175° between two neighbouring piezoelectric patterns with different alignments of the crystalline dipoles (bottom half of Fig. 3g). It reveals that the molecular dipole moments at different polarization states have nearly antiparallel orientations (Fig. 3e shows the PFM imaging of the phase contrast). Thus, the structural deformation and the polarization switching of the polar α-phase P(VDF-TrFE) are simultaneously accomplished by the PFM tip-generated poling field. Besides, a lower phase contrast of ~150° is obtained from the β-phase (the PFM imaging of its phase contrast is illustrated in Fig. 3f).

### Time-dependent piezoelectric behaviours of polar α-phase P(VDF-TrFE) film

A molecular piezoelectric should exhibit a short-term strain recovery and a long-term polarization retention after removing the applied electric field^28^. A superior self-restoration is crucial to the repeatability of the piezoelectric elements; and a reliable electrical polarization offers favourable means of manipulating electromechanical properties for adaptive piezoelectronics.

**Figure 4:**
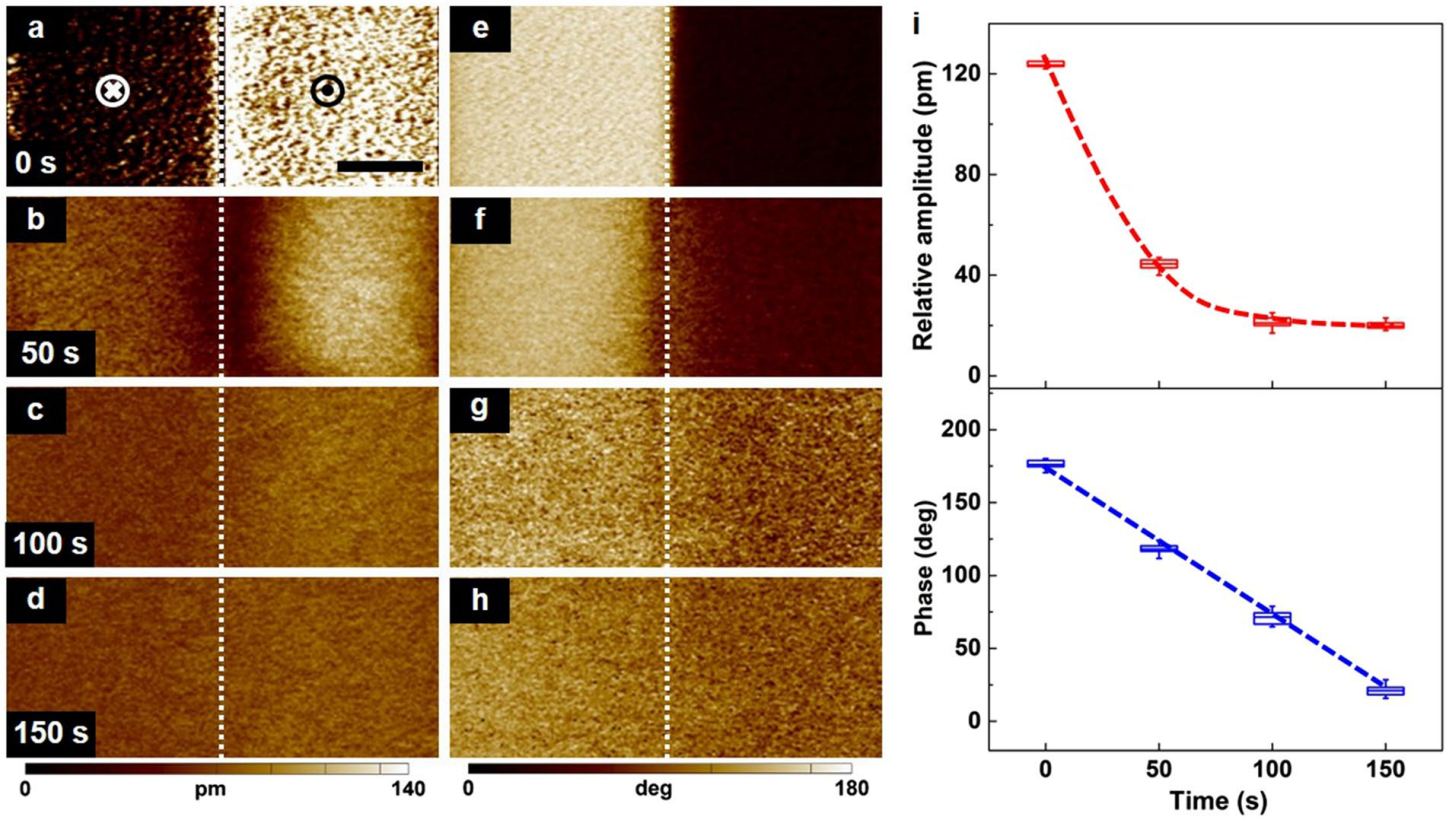
Time-dependent domain piezoelectric behaviours of the ultrathin polar α-phase P(VDF-TrFE) films. (**a–d**) The out-of-plane PFM amplitude images at different time periods. (**e–h**) The out-of-plane PFM phase images at different time periods. All PFM images were acquired over the pre-poled areas. The white dotted lines in (**a–h**) form the boundary between the two adjacent polarized areas with scale bar of 1 μm. (**i**) The phase contrasts and relative amplitudes between two different electrical polarization orientations as a function of the delay times. Blue and red dotted lines are fitting curves to visualize the changing trends.

The two polarization states exhibit distinguishing piezoelectric properties in terms of the mechanical strain and the phase of the poled film^29^. Hence, the relative morphological deformation and the dipole orientation degree should be measured to evaluate the time-dependent nature of a piezoelectric film. The relative amplitude images of the polar α-phase P(VDF-TrFE) are shown in Fig. 4a–d, the mechanical strains rapidly decrease. Meanwhile, the phase contrasts between two different polarization states remain much visible over time (Fig. 4e–h). Figure 4i shows the images obtained for the evolution of the averaged relative amplitude and phase contrast versus different delay times. Initially, the relative amplitude and phase contrast show the maximum values of ~120 pm and ~180°, respectively. It indicates a reliable tortile structure with a polarized molecular chain. When the time is 50 s, the relative amplitude decreases to ~33% of the initial value and the phase contrast maintains a value of ~125°. As the time is further increased from 100 s to 150 s, the relative amplitude presents as a stable value which is comparable to only ~16% of the maximum state. Besides, the phase contrast gradually decreases from ~70° to ~20°. Therefore, the polar α-phase P(VDF-TrFE) films exhibit superior deformation restorability, and the polarization retention time reaches up to ~150 s at zero applied field. It is governed predominantly by the molecular arrangements of the polar α-phase P(VDF-TrFE) and interfacial interactions^16,30^. As a comparison, we also measured the time-dependent piezoelectric behaviours on the β-phase P(VDF-TrFE) films. After removing the electric field, a much smaller maximum mechanical deformation of ~30 pm and a shorter inferior polarization retention of ~75 s are observed (Supplementary Fig. S7).

### Local piezoelectricities of two polarization states

The time-dependence measurements imaged the evolution of the piezoelectric property, which is related to the reorientation of the polarization states. Study on this issue from the property of a respective polarization state is greatly important to exam the origin for such a self-restoration of piezoelectricity. However, for a thick film, it is difficult to measure a small piezoelectric strain due to a relatively large surface roughness; also, a much higher voltage is required to induce a stable piezoelectric polarization, resulting in a severe impediment to the stability and sensitivity of the PFM signals^31^.

**Figure 5:**
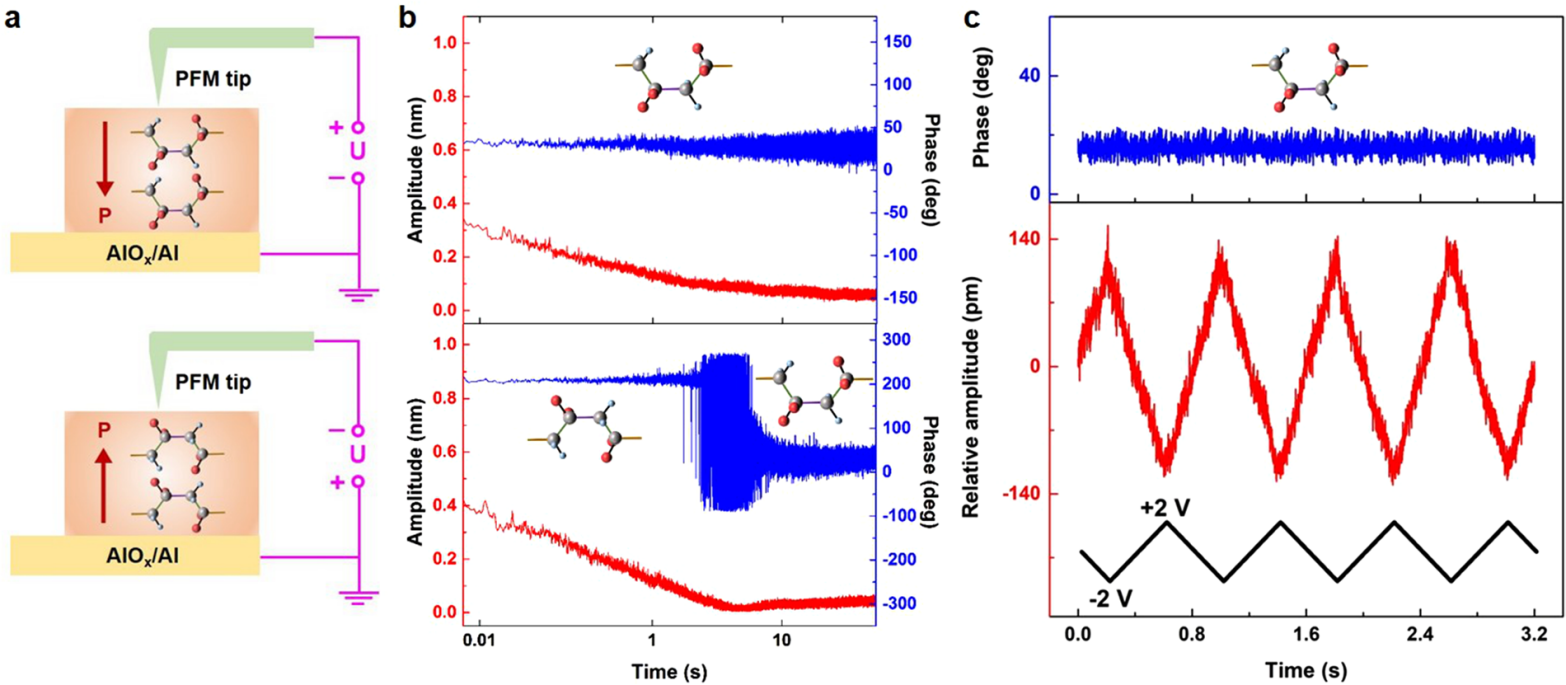
Local piezoelectricities of two polarization states and piezoelectric vibration characteristics. (**a**) Top and bottom panels show the schematics of the downward and upward polarization orientations after positive and negative voltage poling, respectively. (**b**) The phase and amplitude versus the testing time of a downward and an upward polarization orientation. (**c**) The local piezoelectric vibration under an applied low driving voltage (±2 V) while the sample was maintained at a stable downward polarization state.

Thanks to the ultrathin nature of our quasi-two-dimensional P(VDF-TrFE) film, it thus serves as an ideal platform for such a study by *in-situ* probing the piezoelectric signals of each polarization state. As illustrated in Fig. 5a, a conductive PFM tip was directly applied to a bare α-phase P(VDF-TrFE) surface. Then, a dc switching bias pulse of +7 V (or −7 V, pulse duration of 1 s) was generated by the PFM tip to produce a local polarization state (the measured area is ~50 nm in diameter) oriented downward (or upward). The amplitude and phase signals of the corresponding polarization state are presented as a function of time after disconnection of the switching pulse. As shown in Fig. 5b, for the polar α-phase P(VDF-TrFE), the amplitude signals of both polarization orientations notably decrease with increasing testing time and finally exhibit a stable vibrational state. This result is well in line with that of Fig. 4. On the other hand, the phase signals of the downward polarization orientation are stable over time (upper half of Fig. 5b). By contrast, the phase image of the upward polarization orientation changes dramatically. In the bottom half of Fig. 5b, the phase signals of the upward polarization state maintain well for the time of ~3 s, afterwards the phase values start to oscillate. Finally, the phase signals re-emerge as a downward polarization state. This result indicates that the downward polarization is a stable and preferential state, whereas the upward polarization exhibits a polarization relaxation effect^32^. Such a unique feature of our polar α-phase P(VDF-TrFE) is that the electrical polarization tends to relax switch to an energetically preferential orientation when a crystalline film is restricted at a quasi-two-dimensional limit. This feature shows reliable reproducibility in the *in-situ* measurements. Besides, the minor phase vibration observed in Fig. 5b is common due to the crosstalk between the measured signals and the sample topography^31,33^, and the local piezoelectric properties actually exhibit an average effect due to the tip resolution and the grain size.

The intrinsic driving force of the long-range molecular chain rotation is ascribed to a specific variation towards the lowest short-range dipole interaction energy^34,35^. Because a temporary cis-trans conformation, which is associated with an extra steric barrier, is required to undergo in the dipole reversal for a polar α-phase P(VDF-TrFE)^36^, the upward polarization state shows the stabilization time of ~3 s. Moreover, the observed phase oscillation during the conversion from the upward to downward state (3s~8s in bottom half of Fig. 5b) is mainly related to the nucleation and growth of the preferential downward domain^37^. Similar to the polarization behaviours of inorganic piezoelectric ultrathin films, the preferential polarization orientation and the polarization relaxation also indicate the existence of a built-in electric field and a depolarizing field in our asymmetric polar α-phase P(VDF-TrFE)/AlO_x_ structure^38,39^. Besides, the polarization states of the ultrathin β-phase P(VDF-TrFE) exhibit poor endurance (Supplementary Fig. S8).

### Electromechanical coupling properties

To further assess the transformation of the electrical charge to the mechanical strain, we demonstrated the electromechanical coupling behaviours by positioning the conductive PFM tip at a poled point to collect its periodic vibration signals. As illustrated in Fig. 5c, a considerable vibration of a selected point on the poled surface is generated under the excitation of a low triangular wave voltage (the peak voltage and the frequency are ±2 V and 1.25 Hz, respectively), thus revealing a reliable polarization-biased electrostriction of the polar α-P(VDF-TrFE)^40^. Besides, in Supplementary Fig. S9a, a dual-peak vibration with the presence of the polarization reversal is observed at driving voltages exceeding the coercive voltages.

Thus, the electromechanical properties of the polar α-phase P(VDF-TrFE) are evaluated by extracting the local effective piezoelectric coefficient *d*_zz_ and the maximum electric field-induced strain. And *d*_zz_ represents the capability of a piezoactive material to generate the strain along the direction of the applied electric field^1^. The values of *d*_zz_ and the maximum strain of the polar α-phase P(VDF-TrFE) reach up to −46.4 pm V^−1^ and 4.1%, respectively (Fig. 5c, Supplementary Fig. S9b). Both parameters are even higher than those of the β-phase (−41.7 pm V^−1^, 3.4%, as shown in Supplementary Figs S8c and S10b). Besides, the maximum electric field-induced strain of our quasi-two-dimensional polar α-phase P(VDF-TrFE) is one order of magnitude higher than the strain observed in the bulk one^41^ and even comparable to that of ZnO piezoelectric nanowires^3^. It indicates that our quasi-two-dimensional α-phase P(VDF-TrFE) films also yield an improved piezoelectric activity than that of the bulk ones. In our experiment, more than ten individual piezoelectric P(VDF-TrFE) films are fabricated and measured, and typical piezoelectric and crystalline properties are summarized in Table 1.

**Table 1:** Crystalline and piezoelectric properties of ultrathin P(VDF-TrFE) films.

Material	Substrate	Thickness (nm)	Roughness (nm)	*V*_c_ (V)	*d*_zz_ (pm V^−1^)	Local strain (%)	Relative deformation (pm)
α-phase	AlOx/Al	4.0	0.76	+3.7, −3.2	−46.4	4.1	37
β-phase	Au	4.2	1.08	+2.0, −3.6	−41.7	3.4	8

*V*_c_ and *d*_zz_ represent the coercive voltage and the local effective piezoelectric coefficient, respectively.

The electromechanical coupling behaviours of the polar α-phase P(VDF-TrFE) films reveal a large piezoelectric response. Especially, the most important parameter for the evaluation of the piezoelectricity is the local effective piezoelectric coefficient *d*_zz_, which can be expressed as *d*_zz_ ∝ *P*_e_ at low electric fields^26,40^. Thereby, the electrical polarization *P*_e_ is recognized as the key element for the piezoelectric response, which is determined by the value and orientation of the dipole moment^42^. The permanent dipole moment of the polar α-chain P(VDF-TrFE) is 4 × 10^−30^ C m^43^, which is comparable to that of the polar α-PVDF^16^. And the GI-XRD diffractogram exhibits a remarkable intensity for the (110) reflection of the polar α-phase films, manifesting that a considerable number of polymer backbones are oriented parallel to the film plane (Fig. 1i). Thus, the polar α-phase should intrinsically possess a highly oriented electrical polarization. Therefore, the polar α-phase can yield a relatively large value of *d*_zz_. Besides, the piezoelectric deformation in the P(VDF-TrFE), equivalently the cantilever displacement of the PFM, is proportional to the *d*_zz_ value^44^. Thus, the polar α-phase P(VDF-TrFE) can exhibit a high relative morphological deformation, as shown in Fig. 3g.

## Discussion

In summary, we have successfully fabricated quasi-two-dimensional, neat α-phase P(VDF-TrFE) films on the AlO_x_/Al -coated SiO_2_/Si substrates at room temperature. The relative morphological deformation, the local effective piezoelectric coefficient *d*_zz_, and the electric field-induced strain of the polar α-phase P(VDF-TrFE) are 37 pm, −46.4 pm V^−1^, and 4.1%, respectively. The piezoelectric response is even larger than that of the β-phase. The time dependence of polarization-patterned piezoelectric domains, which is related to the piezoelectric properties of two polarization states, is also studied. Our results shed light on the fundamental understanding of the underlying relationship between molecular structures and piezoelectric properties. This will enable new possibilities for prospective electromechanical applications of the piezoactive polar α-phase P(VDF-TrFE), including high-sensitivity micro-displacement detectors, piezoelectric transducers, sonic transmitters and energy harvesters.

## Methods

### Sample preparation

Ultrathin α-phase P(VDF-TrFE) films were deposited using highly doped n-type (100) Si wafers with 50 nm SiO_2_ layers that were sequentially cleaned in an ultrasonic bath in a succession of acetone, isopropanol and deionized water for 10 min each. The bottom electrodes were formed by thermally evaporating Al (10 nm) at a rate of 0.1 Å s^−1^ at a base pressure of 10^−5^ Torr. A native oxidative layer (AlO_x_) was present because of the short exposure to air. P(VDF-TrFE) (70:30 mole ratio, purchased from Solvay, Inc., France) was dissolved in a mixture of DMF and antisolvent *p*-anisaldehyde (~5 mg mL^−1^) at a 0.5 wt.% concentration. A droplet of P(VDF-TrFE) solution was then drop-cast onto the AlO_x_/Al-coated SiO_2_/Si substrate; a mechanical pump with a pumping speed of ~7 L min^−1^ was used to vent the air through a pipe positioned ~1 mm from the upper surface of the droplet. Pristine films with no post-annealing treatment were used for characterization. The film preparation was performed at room temperature in a glove box under high-purity N_2_ conditions.

### Film characterization

A Keyence VHX-5000 digital microscope (Keyence Ltd. Japan) was used to obtain the optical microscopy images. The crystal phase of the P(VDF-TrFE) films was characterized by micro-Raman spectroscopy on a NanofindeNr FLEX Raman confocal Microscope (Tokyo Instruments, Inc., Japan) with 532 nm laser excitation. A Rigaku Smartlab X-ray diffractometer operated at 3 kW X-ray power was used to perform the GI-XRD to further assess the crystalline properties of the α-phase P(VDF-TrFE) films. Regular AFM characterizations were performed under ambient conditions on a Veeco Multimode 8 and SPA-400 scanning probe microscope controlled by an SPI 4000 probe station (Seiko Instruments, Inc., Japan). The piezoelectric hysteresis loops, domain piezoelectric behaviours, local piezoelectricities of polarization states, and electromechanical coupling properties were measured using an Asylum Research Cypher scanning probe microscope (Asylum Research, Oxford Instruments, China). Nanosensors PPP-EFM chromium/platinum-iridium (Cr/Pt-Ir)-coated silicon cantilevers (radius of ~25 nm) were used for the PFM measurements. The force constant of the cantilever was 3 N m^−1^, and its resonant frequency in air was approximately 70 kHz. The in-contact resonant frequency on the ultrathin P(VDF-TrFE) was in the range from 320 to 350 kHz. The hysteresis loops versus voltage were collected in the dual alternating current resonance tracking (DART) mode with a triangle pulse of 7.0 V applied to the tip. The domains were written onto the P(VDF-TrFE) surface in lithography mode, and the PFM images were acquired over the poled areas. The bottom AlO_x_/Al substrate was electrically grounded for the PFM measurements.

## Electronic supplementary material

Supplementary information

## References

[1] You Y (2017). An organic-inorganic perovskite ferroelectric with large piezoelectric response. Science.

[2] Saito Y (2004). Lead-free piezoceramics. Nature.

[3] Wang Z, Song L (2006). Piezoelectric Nanogenerators Based on Zinc Oxide Nanowire Arrays. Science.

[4] Furukawa T (1989). Ferroelectric properties of vinylidene fluoride copolymers. Phase Transit..

[5] Legrand JF (1989). Structure and ferroelectric properties of P(VDF-TrFE) copolymers. Ferroelectrics.

[6] Ohigashi H (1984). Piezoelectric and ferroelectric properties of P(VDF-TrFE) copolymers and their application to ultrasonic transducers. Ferroelectrics.

[7] Sharma T (2012). Patterning piezoelectric thin film PVDF-TrFE based pressure sensor for catheter application. Sensor Actuat. A Phys..

[8] Persano L (2013). High performance piezoelectric devices based on aligned arrays of nanofibers of poly(vinylidenefluoride-co-trifluoroethylene). Nat. Commun..

[9] Chang C (2010). Direct-Write Piezoelectric Polymeric Nanogenerator with High Energy Conversion Efficiency. Nano Lett..

[10] Choi Y-Y (2015). Enhancement of Local Piezoresponse in Polymer Ferroelectrics via Nanoscale Control of Microstructure. ACS Nano..

[11] Liu Y (2010). Rapid Nanoimprinting and Excellent Piezoresponse of Polymeric Ferroelectric Nanostructures. ACS Nano..

[12] Tashiro K, Kobayashi M (1989). Structural phase transition in ferroelectric fluorine polymers: X-ray diffraction and infrared/Raman spectroscopic study. Phase Transitions..

[13] Davis GT (1978). Electric-field-induced phase changes in poly(vinylidene fluoride). J. Appl. Phys..

[14] Naegele D (1978). Formation of a new crystal form α_p_ of poly(vinylidene fluoride) under electric-field. Macromolecules.

[15] Bachmann M (1980). The crystal structure of phase-IV of poly(vinylidene fluoride). J. Appl. Phys..

[16] Li M (2013). Revisiting the δ-phase of poly(vinylidene fluoride) for solution-processed ferroelectric thin films. Nat. Mater..

[17] Sussner H (1979). The piezoelectric polymer PVF_2_ and its applications. Ultrasonics Symposium..

[18] Lei C (2014). Influence of Room-Temperature-Stretching Technology on the Crystalline Morphology and Microstructure of PVDF Hard Elastic Film. J. Appl. Polym. Sci..

[19] Xu H (2000). Structural, Conformational, and Polarization Changes of Poly(vinylidene fluoride-trifluoroethylene) Copolymer Induced by High-Energy Electron Irradiation. Macromolecules.

[20] Wang Q (2016). 2D Single-Crystalline Molecular Semiconductors with Precise Layer Definition Achieved by Floating-Coffee-Ring-Driven Assembly. Adv. Func. Mater..

[21] Song L (2017). Speed up Ferroelectric Organic Transistor Memories by Using Two-Dimensional Molecular CrystallineSemiconductors. ACS Appl. Mater. Interfaces.

[22] Yuan S (2011). Ferroelectricity of ultrathin ferroelectric Langmuir–Blodgett polymer films on conductive LaNiO_3_ electrodes. Mater. Lett..

[23] Gil HAC (1998). Structural Modifications of Vinylidene Fluoride-Trifluoroethylene (70–30) Copolymer Induced by X-ray Irradiation. Polym. Degrad. Stab..

[24] Gregorio R (2006). Determination of the α, β, and γ Crystalline Phases of Poly(vinylidene fluoride) Films Prepared at Different Conditions. J. Appl. Polym. Sci..

[25] Farmer BL (1972). Polymorphism of poly(vinylidene fluoride): potential energy calculations of the effects of head‐to‐head units on the chain conformation and packing of poly(vinylidene fluoride). J. Appl. Phys..

[26] Katsouras I (2016). The negative piezoelectric effect of the ferroelectric polymer poly(vinylidene fluoride). Nat. Mater..

[27] Lindner M (2004). Charged cellular polymers with “ferroelectretic” behavior. IEEE Trans. Diel. Electr. Insul..

[28] Valasek J (1921). Piezoelectricity and applied phenomenon in Rochelle salt. Phys. Rev..

[29] Birk H (1991). The local piezoelectricity of thin polymer films observed by scanning tunnelling microscopy. J. Vac. Sci. Technol. B.

[30] Eberle G (1991). Polarization dynamics of VDF-TrFE copolymers. IEEE Trans. Electr. Insul..

[31] Rodriguez BJ (2007). Dual-frequency resonance-tracking atomic force microscopy. Nanotechnology..

[32] Kim DJ (2005). Polarization Relaxation Induced by a Depolarization Field in Ultrathin Ferroelectric BaTiO_3_ Capacitors. Phys. Rev. Lett..

[33] Harnagea C (2003). Contact resonances in voltage-modulated force microscopy. Appl. Phys. Lett..

[34] Guo D (2013). Impact of confinement-induced cooperative molecular orientation change on the ferroelectric size effect in ultrathin P(VDF-TrFE) films. Macromolecules.

[35] Duan CG (2004). Simulations of ferroelectric polymer film polarization: The role of dipole interactions. Phys. Rev. B..

[36] Lovinger AJ (1981). Molecular mechanism for alpha-delta transformation in electrically poled poly(vinylidene fluoride). Macromolecules.

[37] Hu W (2014). Universal Ferroelectric Switching Dynamics of Vinylidene Fluoride-trifluoroethylene Copolymer Films. Sci Rep..

[38] Lichtensteiger C (2014). Tuning of the Depolarization Field and Nanodomain Structure in Ferroelectric Thin Films. Nano Lett..

[39] Tagantsev AK, Gerra G (2006). Interface-induced phenomena in polarization response of ferroelectric thin films. J. Appl. Phys..

[40] Furukawa T, Seo N (1990). Electrostriction as the Origin of Piezoelectricity in Ferroelectric Polymers. Jpn. J. Appl. Phys..

[41] Cheng Z-Y (2001). Electrostrictive poly(vinylidene fluoride-trifluoroethylene) copolymers. Sens. Actuators, A.

[42] Devonshire AF (1951). Theory of barium titanate. II Philos. Mag..

[43] Wang Z-Y (2007). Structure, phase transition and electric properties of poly(vinylidene fluoride-trifluoroethylene) copolymer studied with density functional theory. Polymer.

[44] Gruverman A, Kalinin SV (2006). Piezoresponse force microscopy and recent advances in nanoscale studies of ferroelectrics. J. Mater. Sci..

