# Supplementary material for: Unveiling the piezoelectric nature of polar α-phase P(VDF-TrFE) at quasi-two-dimensional limit
Source: Sci Rep. 2018 Jan 11;8:532. doi: 10.1038/s41598-017-18845-2 (PMC5765141; doi:10.1038/s41598-017-18845-2)
Supplement: Supplementary file 1 — Supplementary information [file 41598_2017_18845_MOESM1_ESM.pdf]

Supplementary Information

Unveiling the piezoelectric nature of polar α-phase P(VDF-TrFE) at quasi-two-dimensional limit

Jun Qian[1], Sai Jiang[1], Qijing Wang[1], Shushu Zheng[2], Shuya Guo[1], Chang Yi[3], Jianpu Wang[3], Xinran Wang[1], Kazuhito Tsukagoshi[2], Yi Shi[1] and Yun Li[1]

[1]National Laboratory of Solid-State Microstructures, School of Electronic Science and Engineering, Collaborative Innovation Center of Advanced Microstructures, Nanjing University, Nanjing 210093, P. R. China. [2]International Center for Materials Nanoarchitectonics (WPI-MANA), National Institute for Materials Science (NIMS), Tsukuba, Ibaraki 305-0044, Japan. [3]Key Laboratory of Flexible Electronics and Institute of Advanced Materials, Jiangsu National Synergistic Innovation Center for Advanced Materials, Nanjing Tech University, Nanjing 211816, P. R. China. Correspondence and requests for materials should be addressed to Y.L. (e-mail: yli@nju.edu.cn) or K.T. (email: TSUKAGOSHI.Kazuhito@nims.go.jp) or Y. S. (email: yshi@nju.edu.cn).

Supplementary Figures

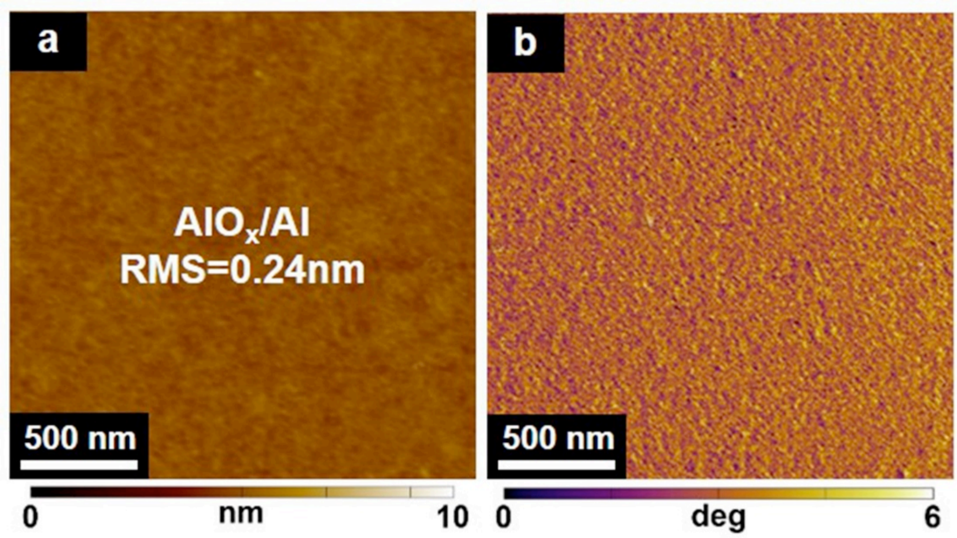

Supplementary Figure 1. Morphological properties of the AlO_x/Al substrate. (**a**) AFM topography image of the AlO_x/Al surface with a root-mean-square (RMS) roughness of 0.24 nm. (**b**) The AFM phase image of the AlO_x/Al surface.

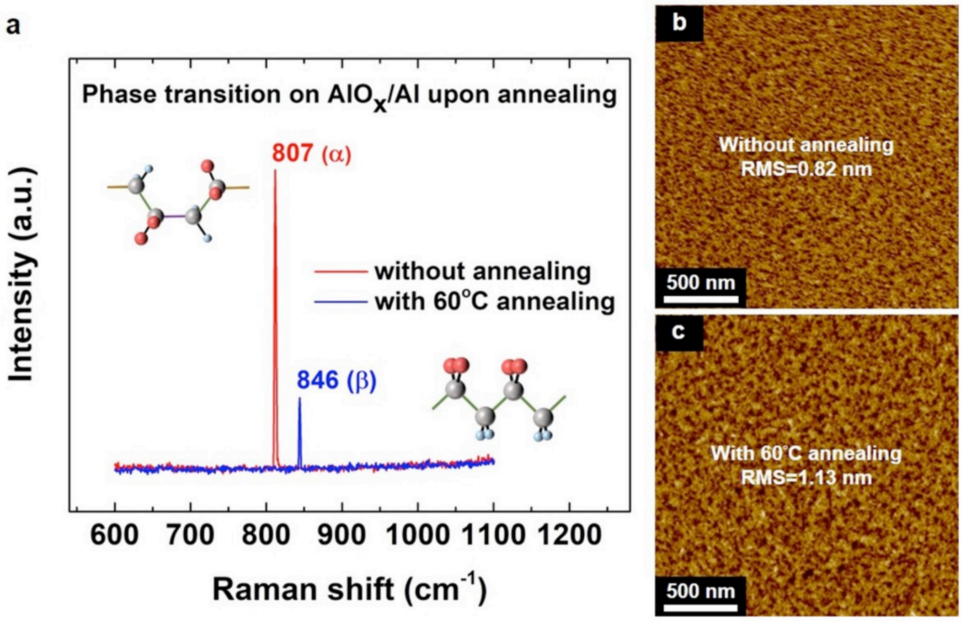

Supplementary Figure 2. Phase transition of the ultrathin P(VDF-TrFE) films on AlO_x/Al after annealing. (**a**) Raman spectra of the ultrathin P(VDF-TrFE) films on AlO_x/Al before and after thermal annealing. The sample was placed on a hotplate for thermal annealing at 60 °C for 10 min. After thermal annealing, the Raman peak shifts from 807 cm^{-1} (α-phase) to 846 cm^{-1} (β-phase), thus indicating that the thermal annealing induces recrystallization of the ultrathin pristine α-phase films. Specifically, the α-P(VDF-TrFE) is a metastable phase under ambient conditions. (**b**,**c**) The AFM morphological images of the ultrathin P(VDF-TrFE) films on AlO_x/Al (**b**) before and (**c**) after the thermal annealing, which show an increase in surface roughness, owing to the thermal annealing.

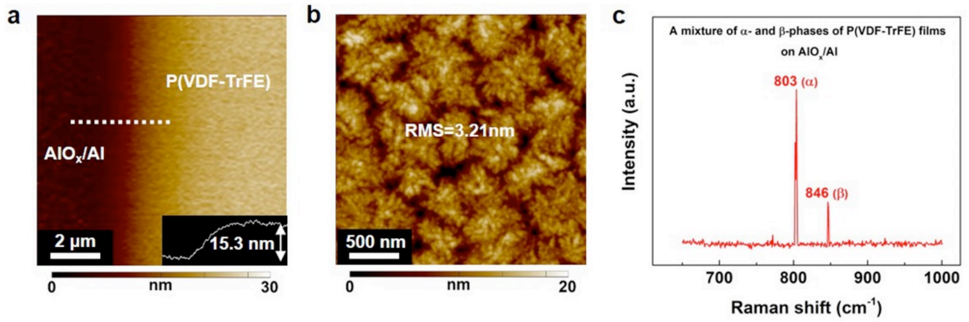

Supplementary Figure 3. (**a**) AFM height image of a P(VDF-TrFE) film with the thickness of 15.3 nm. (**b**) AFM surface topography of a P(VDF-TrFE) film with the RMS roughness of 3.21 nm. (**c**) Raman spectra of the sample, clearly showing the signals of the α- and β-phase. Thermodynamically, the most stable phase is the β-phase for P(VDF-TrFE). As the film thickness was increased, the presence of the TrFE monomers P(VDF-TrFE) destabilized the α structure. Therefore, P(VDF-TrFE) films appeared to contain two phases.

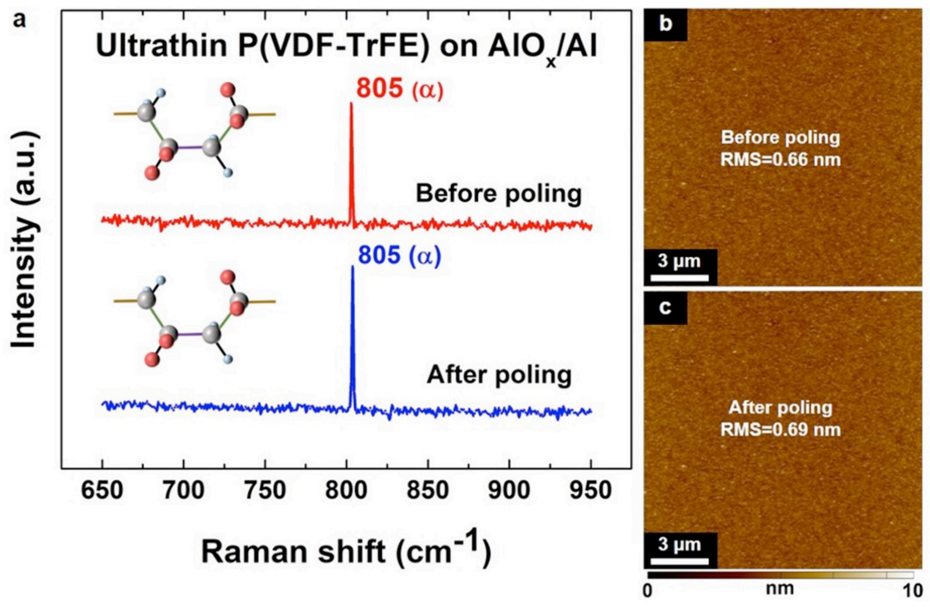

Supplementary Figure 4. (**a**) Raman spectra of the ultrathin P(VDF-TrFE) films on AlO_x/Al before and after the poling. A PFM writing process was used as an electric poling for the α-phase P(VDF-TrFE) film (the maximum writing voltage was set to 7 V). No Raman signals of the β- and the γ-phase within the scanning microdomain are observed after application of the PFM writing. Thus, our α-phase P(VDF-TrFE) films are stable under the electrical poling, and the PFM writing only leads to the formation of polar α-phase P(VDF-TrFE). Besides, the obtained piezoelectric hysteresis loops (Figure 2) also indicates that the polarization switching and the piezoelectric strain of our polar α-phase P(VDF-TrFE) were simultaneously accomplished by a PFM tip-generated poling field. (**b**,**c**) The AFM morphological image of ultrathin P(VDF-TrFE) films on AlO_x/Al (**b**) before and (**c**) after the poling. The surface roughness values are nearly identical, thus indicating the topography of the poled area is not affected by the writing process.

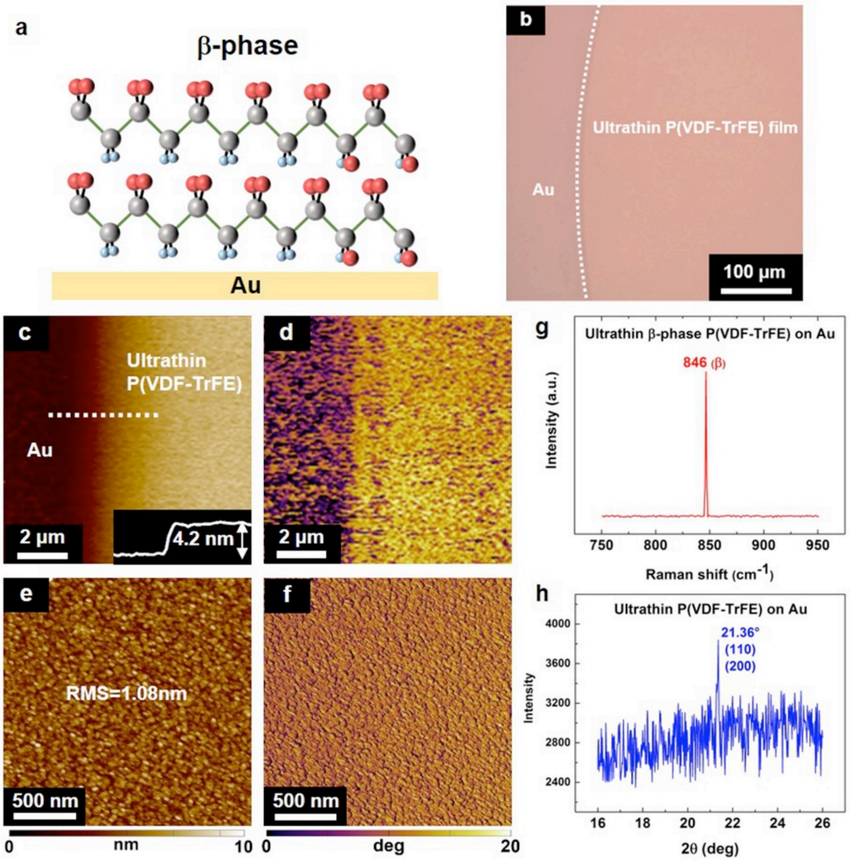

Supplementary Figure 5. The fabrication and crystalline properties of the ultrathin β-phase P(VDF-TrFE) films. (**a**) A schematic of the molecular conformation of the β-phase P(VDF-TrFE) deposited on Au. (**b**) Optical micrograph of the P(VDF-TrFE) film. (**c**,**d**) AFM height and phase images of a P(VDF-TrFE) film. (**e**,**f**) AFM surface topography and phase images of a P(VDF-TrFE) film. (**g**) Raman spectra of the sample in Fig. 1c, clearly showing the signal of the β-phase. (**h**) The 2θ scan image taken from the GI-XRD measurement. A typical (110, 200) reflection is obtained, thus indicating that ultrathin P(VDF-TrFE) films on Au are composed of an all-trans β-phase.

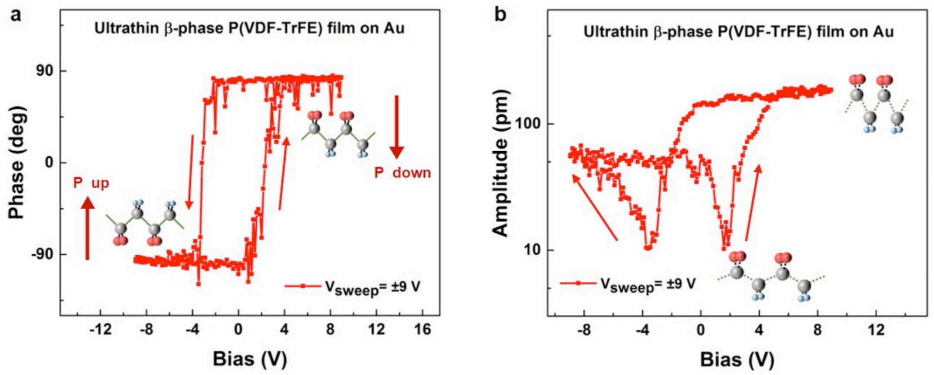

Supplementary Figure 6. Piezoelectric hysteresis loops of the quasi-two-dimensional β-phase P(VDF-TrFE) films on Au substrates. (**a**) The local PFM phase curves with an 180° phase contrast are observed. The insets show the molecular structures corresponding to the two polarization states. (**b**) The local PFM amplitude curves with the expected classical butterfly shape. The insets reveal the stretched and compressed configurations of the β-phase P(VDF-TrFE). The hysteresis loops are recorded with a sweep voltage of ±9 V; the arrows indicate the sweep direction.

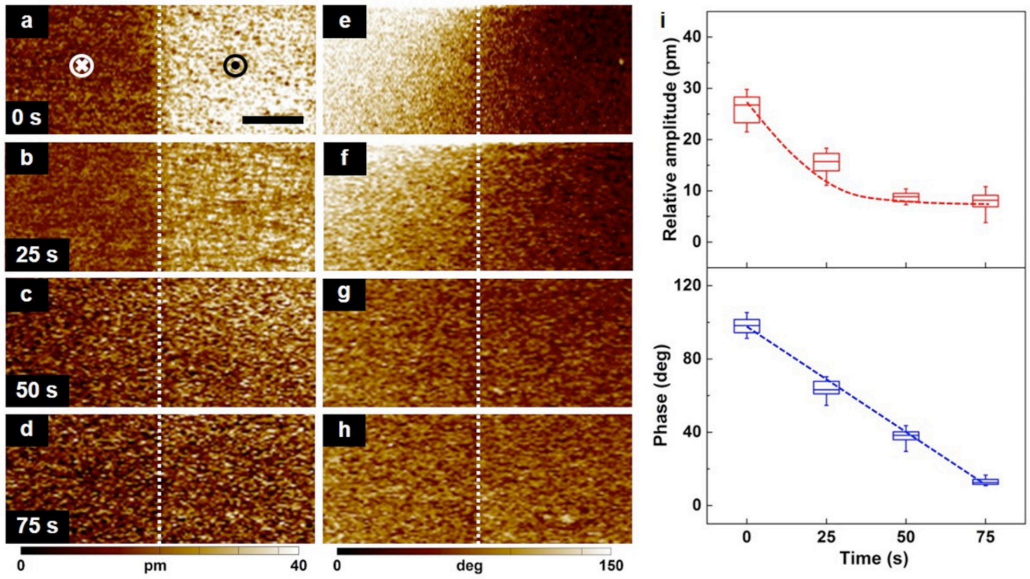

Supplementary Figure 7. Time-dependent piezoelectric behaviours of the ultrathin β-phase P(VDF-TrFE) films. (**a-d**) The out-of-plane PFM amplitude images at different time periods. (**e-h**) The out-of-plane PFM phase images at different time periods. All PFM images were acquired over the pre-poled areas. The white dotted lines in (**a-h**) form the boundary between the two adjacent polarized areas with scale bar of 1 μm. (**i**) The phase contrasts and relative amplitudes between two different electrical polarization orientations as a function of the delay times. Blue and red dotted lines are fitting curves to visualize the changing trends.

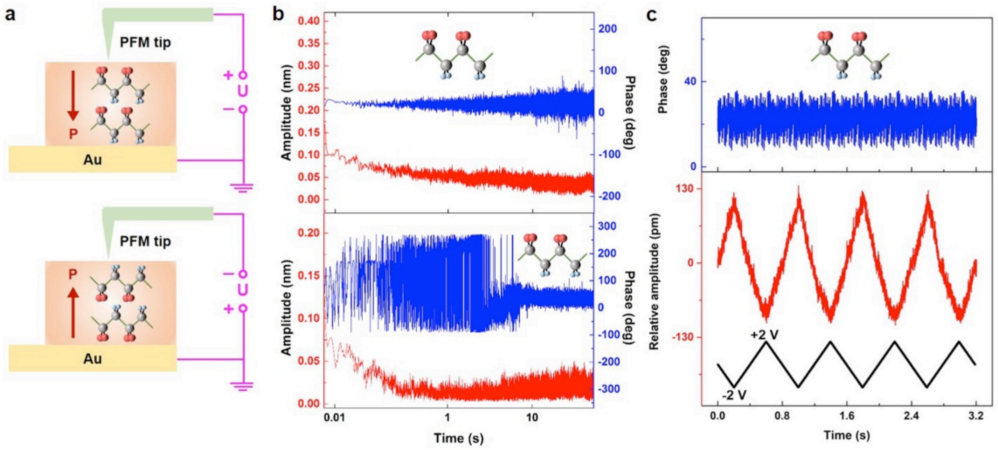

Supplementary Figure 8. Local piezoelectricities of two polarization states and piezoelectric vibration characteristics of the ultrathin β-phase P(VDF-TrFE) films. (**a**) Top and bottom panels show the schematics of the downward and upward polarization orientations after positive and negative voltage poling, respectively. (**b**) The phase and amplitude versus the testing time of a downward and an upward polarization orientation. (**c**) The local piezoelectric vibration under an applied low driving voltage (±2 V), and the sample is maintained at a stable downward polarization state.

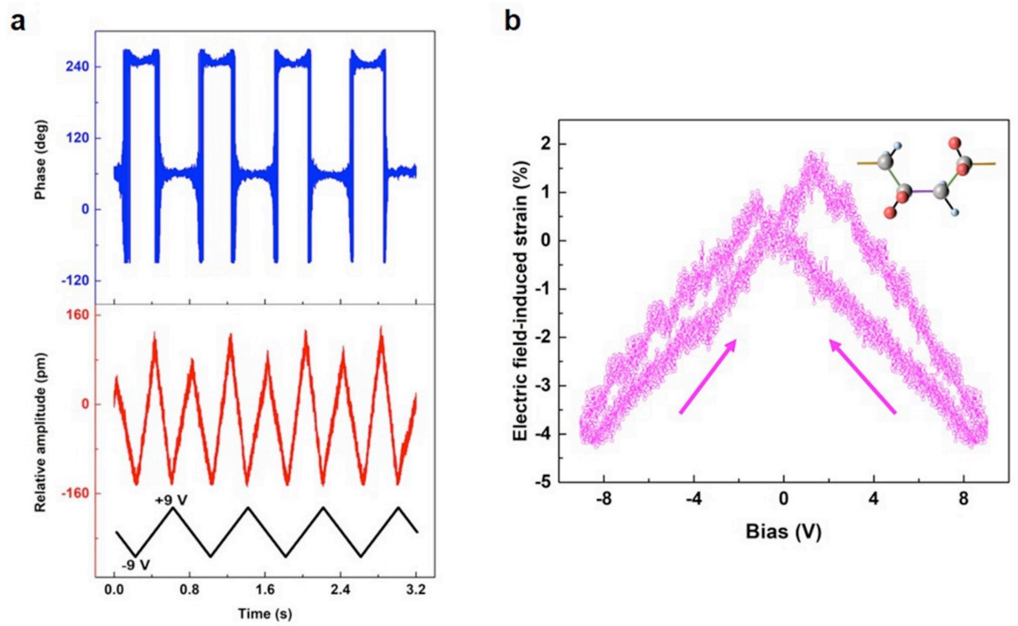

Supplementary Figure 9. Local piezoelectric vibration measurements at a high driving voltage for the quasi-two-dimensional polar α-phase P(VDF-TrFE) films. (**a**) The phase and relative amplitude at a high driving voltage exceeding the coercive field versus testing time. The peak voltage and frequency of the applied driving voltage are ±9 V and 1.25 Hz, respectively. Within a testing cycle, the existence of two vibration peaks and the polarization reversal is observed. (**b**) The electric field-induced strain as a function of the external voltage. The inset map shows the molecular structure of the α-phase; the arrows indicate the sweep direction.

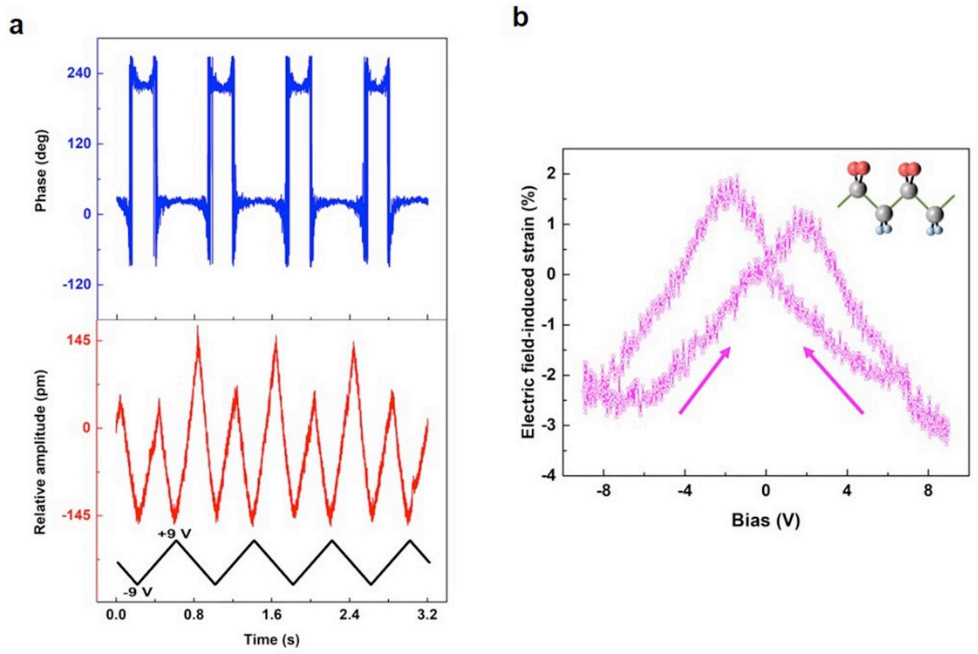

Supplementary Figure 10. Local piezoelectric vibration measurements at a high driving voltage for the quasi-two-dimensional β-phase P(VDF-TrFE) films. (**a**) The phase and relative amplitude at a high driving voltage exceeding the coercive field versus testing time. The peak voltage and frequency of the applied driving voltage are ±9 V and 1.25 Hz, respectively. Within a testing cycle, the existence of two vibration peaks and the polarization reversal is observed. (**b**) The electric field-induced strain as a function of the external voltage. The inset map shows the molecular structure of the β-phase; the arrows indicate the sweep direction.
